# Epidermal Growth Factor Receptor Signaling Enhances the Proinflammatory Effects of *Staphylococcus aureus* Gamma-Toxin on the Mucosa

**DOI:** 10.3390/toxins9070202

**Published:** 2017-06-28

**Authors:** Aaron N. Gillman, Laura M. Breshears, Charles K. Kistler, Patrick M. Finnegan, Victor J. Torres, Patrick M. Schlievert, Marnie L. Peterson

**Affiliations:** 1 Department of Pharmacy, University of Tromsø, Tromsø 9019, Troms, Norway; aaron.gillman@uit.no; 2 Department of Experimental and Clinical Pharmacology, University of Minnesota, Minneapolis, MN 55455, USA; bresh006@umn.edu; 3 Extherid Biosciences, LLC., Jackson, WY 83001, USA; charles@extherid.com; 4 School of Pharmacy, University of Wyoming, Laramie, WY 82071, USA; Patrick.Finnegan@uwyo.edu; 5 Department of Microbiology, New York University School of Medicine, New York, NY 10016, USA; victor.torres@nyumc.org; 6 Department of Microbiology, University of Iowa Carver College of Medicine, Iowa City, IA 52242, USA; patrick-schlievert@uiowa.edu

**Keywords:** *Staphylococcus aureus*, gamma-toxin, epidermal growth factor receptor, menstrual toxic shock syndrome, tyrosine kinase inhibitors, and mucosal immune response

## Abstract

*Staphylococcus aureus* (*S. aureus*) produces many different exotoxins including the gamma-toxins, HlgAB and HlgCB. Gamma-toxins form pores in both leukocyte and erythrocyte membranes, resulting in cell lysis. The genes encoding gamma-toxins are present in most strains of *S. aureus,* and are commonly expressed in clinical isolates recovered from menstrual Toxic Shock Syndrome (mTSS) patients. This study set out to investigate the cytotoxic and proinflammatory effects of gamma-toxins on vaginal epithelial surfaces. We found that both HlgAB and HlgCB were cytotoxic to cultured human vaginal epithelial cells (HVECs) and induced cytokine production at sub-cytotoxic doses. Cytokine production induced by gamma-toxin treatment of HVECs was found to involve epidermal growth factor receptor (EGFR) signaling and mediated by shedding of EGFR ligands from the cell surface. The gamma-toxin subunits displayed differential binding to HVECs (HlgA 93%, HlgB 97% and HlgC 28%) with both components (HlgAB or HlgCB) required for maximum detectable binding and significant stimulation of cytokine production. In studies using full thickness ex vivo porcine vaginal mucosa, HlgAB or HlgCB stimulated a dose-dependent cytokine response, which was reduced significantly by inhibition of EGFR signaling. The effects of gamma-toxins on porcine vaginal tissue and cultured HVECs were validated using ex vivo human ectocervical tissue. Collectively, these studies have identified the EGFR-signaling pathway as a key component in gamma-toxin-induced proinflammatory changes at epithelial surfaces and highlight a potential therapeutic target to diminish toxigenic effects of *S. aureus* infections.

## 1. Introduction

*Staphylococcus aureus* is a diverse pathogen that is capable of infecting many human tissues and organs causing a wide range of illnesses including skin and soft tissue infections, pneumonia, necrotizing fasciitis and endocarditis [1]. The opportunity for *S. aureus* to cause such a broad spectrum of clinical conditions is related undoubtedly to widespread asymptomatic colonization of the nares, axillae, skin and vagina [1]. *S. aureus* contributes to disease through production of an arsenal of virulence factors including secreted toxins, which disrupt the host immune response. These secreted toxins include superantigens (SAgs) such as toxic shock syndrome toxin-1 (TSST-1), and cytolysins, such as alpha-toxin and gamma-toxins. *S. aureus* causes the systemic disease, toxic shock syndrome (TSS) through the activity of SAgs. About half of TSS cases are associated with menstruation (menstrual, mTSS) and occur in the absence of significant bacteremia [2,3]. The cytolysins of *S. aureus* have been characterized primarily as hemolysins and leukocidins [4,5]. Previous studies have linked cytolysins to the pathogenesis of *S. aureus* in murine bacteremia and septic arthritis and demonstrated lysis of human neutrophils, macrophages and red blood cells [6,7,8,9]. However, there is evidence that they may contribute to mTSS progression through enhancement of local inflammation and disruption of the epithelial barrier, increasing SAg penetration into the mucosa [10,11]. 

The primary mediator of mTSS is TSST-1, which is the only SAg capable of causing disease from the vaginal mucosa in animal models [12]. However, TSST-1 must penetrate the epithelium to gain access to its primary targets, T-cells and antigen presenting cells, to cause mTSS. While TSST-1 can flux across the vaginal mucosa independently, penetration through tissue is enhanced when epithelial integrity is compromised [11]. Disruption of epithelial integrity can result from direct injury through cell lysis or as a result of inflammation. Independently, TSST-1 can induce proinflammatory cytokines in human vaginal epithelial cells (HVECs) through activation of (a) disintegrin and metalloproteinases 10 and 17 (ADAM-10 and -17) resulting in shedding of epidermal growth factor receptor (EGFR) ligands and subsequent activation of the EGFR [13,14]. While EGFR signaling is strongly associated with homeostasis and growth of epithelial cells, the EGFR signaling pathway is also a component of the innate immune response to injury [14]. The *S. aureus*-encoded cytolysin, alpha-toxin, is known to disrupt the vaginal mucosa, increase TSST-1 penetration across tissue, and enhance TSST-1-induced cytokine production in HVECs [11]. However, alpha-toxin is likely to play a role in only a subset of mTSS cases as it is produced by less than 20% of mTSS clinical isolates [15]. The closely related gamma toxins may play a larger role in mTSS progression as they are produced by all clinical isolates tested, yet their effects on the vaginal mucosa are unknown. In this study, we examined the binding and signaling capabilities of the gamma-toxin cytolysins on female reproductive tract cells and tissues.

The *S. aureus* gamma-toxins are β-barrel pore-forming toxins that are secreted from the bacteria as monomers. The monomeric subunits insert into target cell membranes, oligomerize, and form pores. This activity causes cation efflux, osmotic imbalance and cell lysis [4]. The gamma-toxin monomers are comprised of S and F class subunits, corresponding to slow and fast elution from an ion exchange column [5]. Mature gamma-toxin pores are composed of 1:1 ratios of one F component (HlgB) oligomerized with one of the two S class subunits, either the A subunit (HlgA) or the C subunit (HlgC) [8,16]. Thus, two distinct toxins are produced, HlgAB or HlgCB. The gamma-toxin genes (*hlgA*, *hlgB* and *hlgC*) are the most up-regulated cytolysins transcribed when *S. aureus* is exposed to blood, and are present in the majority of examined strains (99%) [6,17]. However, despite widespread prevalence and strong transcriptional induction after exposure to blood, little is known about the mechanisms of interaction between gamma-toxins and the vaginal epithelium, a mucosal surface commonly colonized by *S. aureus* and regularly exposed to blood.

In this study, the effects of the gamma-toxins, HlgAB and HlgCB, on the vaginal epithelium were investigated. We hypothesized that gamma-toxins have pathogenic activity at the vaginal mucosa through direct cytotoxic and proinflammatory actions mediated by EGFR signaling. We found that HlgAB and HlgCB were cytotoxic to immortalized human vaginal epithelial cells (HVECs) and induced production of proinflammatory cytokines at sub-cytolytic doses. Gamma-toxin-induced cytokine production in HVECs was determined to involve EGFR-signaling, mediated by shedding of the EGFR ligands. We determined that Hlg A, B, C toxin subunits bound HVECs. We confirmed the presence of known gamma-toxin receptors, CXCR1, CXCR2, and CCR2 (HlgA) and C5aR (HlgC), in HVECS with CXCR1 having the highest relative expression and C5aR the lowest expression [18]. Additionally, in ex vivo full thickness porcine vaginal mucosa (PVM) (a model for human vaginal mucosa), and ex vivo human ectocervical tissue, gamma-toxins (HlgAB and HlgCB) stimulated a dose-dependent IL-8 response that was reduced significantly by the addition of an EGFR-specific tyrosine kinase inhibitor, AG1478. Overall, our data support our hypothesis and suggest a role for gamma-toxins in *S. aureus*-induced inflammation at the vaginal mucosa.

## 2. Results

### 2.1. Gamma-Toxins Are Cytotoxic and Proinflammatory to HVECs

**Figure 1:**
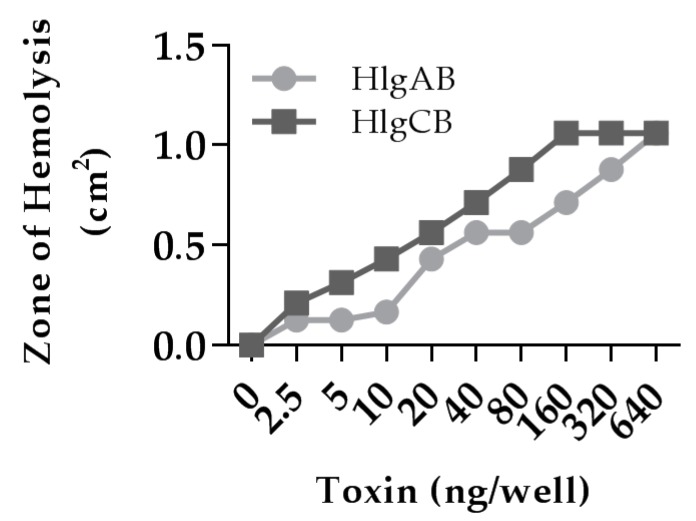
Gamma-toxin is hemolytic to rabbit erythrocytes. 100 μL of gamma-toxin at a 1:1 molar ratio in Tris-Buffered Saline (TBS) at the indicated dose was placed in 0.3 cm diameter wells within 0.4% agar containing 5% rabbit erythrocytes for 4 h and the zone of hemolysis was measured. Data represent area from individual wells.

Gamma-toxins are hemolytic to rabbit erythrocytes [8]. The biological activity of our purified recombinant gamma-toxins was comparable to previously reported activities and showed dose-dependent hemolysis of rabbit erythrocytes in soft agar in the nanogram range (Figure 1).

**Figure 2:**
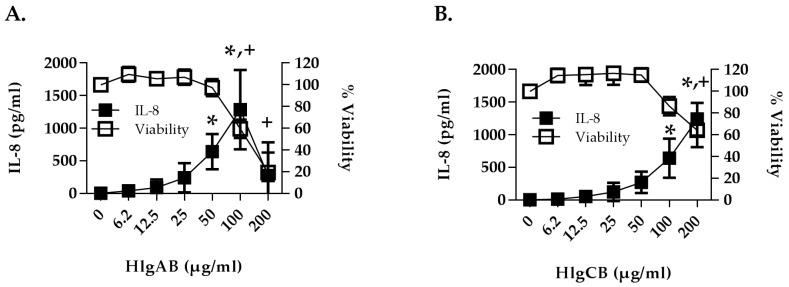

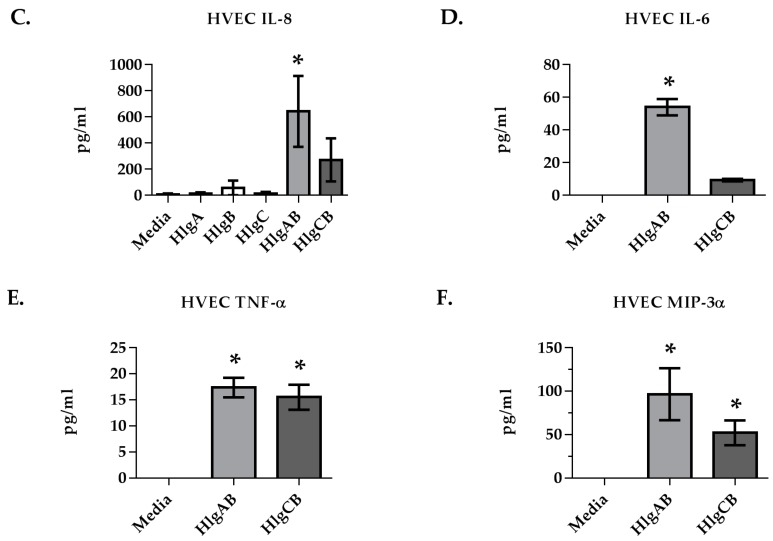
Gamma-toxin is cytotoxic and induces production of proinflammatory cytokines in human vaginal epithelial cells (HVECs). HVECs were exposed to (**A**) HlgAB or (**B**) HlgCB at the indicated doses for 6 h and processed for cytotoxicity (open squares) by MTT assay or cytokine production (filled squares) by ELISA. For A and B, asterisks (IL-8) and crosses (viability) indicate significant changes (*p* < 0.05) from media alone controls; (**C**) IL-8; (**D**) IL-6; (**E**) TNF-α and (**F**) MIP-3α production from HVECs treated with individual subunits of gamma-toxin (50 µg/mL) or HlgAB and HlgCB (50 µg/mL) at a 1:1 molar ratio. Data show mean +/− standard deviation. For D–F, asterisks indicate a significant difference from media alone controls (*p* < 0.05).

The potential interactions of gamma-toxins with the female reproductive tract were first investigated using HVECs. Gamma-toxins (HlgAB and HlgCB) were determined to be both proinflammatory and cytotoxic to HVECs. HlgAB and HlgCB stimulated a dose-dependent IL-8 response in HVECs that was significant at 50 µg/mL and 100 µg/mL, respectively (Figure 2A,B). Toxins were cytotoxic at 100 µg/mL for HlgAB and 200 µg/mL for HlgCB, and at 200 µg/mL HlgAB reduced cell viability by ~79% and HlgCB by ~36%. Individual subunits (HlgA, HlgB and HlgC) at equivalent doses (50 µg/mL) were not proinflammatory (Figure 2C). When compared at sub-cytotoxic doses (50 µg/mL), HlgAB stimulated production of IL-6 and both toxins stimulated production of TNF-α and MIP-3α with HlgAB being more potent than HlgCB (Figure 2D–F). These data demonstrate that gamma toxins (HlgAB and HlgCB) are proinflammatory and cytotoxic to the epithelial cells of the vaginal mucosa.

### 2.2. Gamma-Toxins Stimulation of IL-8 from HVECs Is Enhanced by EGFR Signaling and Involves Shedding of EGFR Ligands

**Figure 3:**
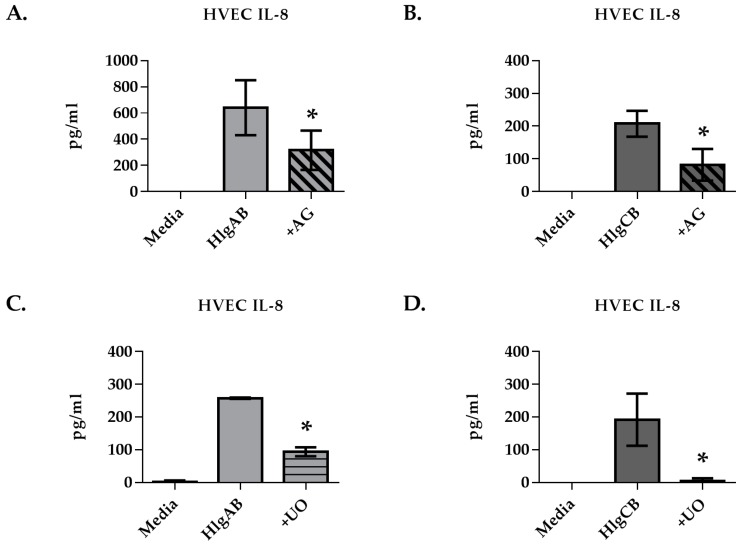
Epidermal growth factor receptor (EGFR) inhibition reduces significantly gamma-toxin-induced IL-8 production in HVECs. HVECs were exposed to HlgAB (50 µg/mL) or HlgCB (100 µg/mL) at a 1:1 molar ratio for 6 h and IL-8 production was analyzed by ELISA. IL-8 production in response to HlgAB or HlgCB was attenuated in the presence of (**A**,**B**) AG1478 or (**C**,**D**) UO126. Data show mean +/− standard deviation. Asterisks indicate significant difference from gamma-toxin treated cells with no inhibitor present (*p* < 0.05).

We next investigated the potential mechanism(s) by which HlgAB and HlgCB induced IL-8 from HVECs. IL-8 was used as a biomarker to characterize the cellular response of HVECs to gamma-toxin due to robust production of this cytokine in response to both toxins (HlgAB 50 µg/mL and HlgCB 100 µg/mL). Given that the exotoxins, TSST-1 and α-toxin, both stimulate EGFR signaling in epithelial cells, we hypothesized that the EGFR may also play a role in the response to gamma-toxin [13,19]. The EGFR inhibitor AG1478 reduced significantly the IL-8 response of HVECs to HlgAB and HlgCB greater than 2-fold (Figure 3A,B). Since mitogen-activated protein (MAP) kinase is a downstream effector of EGFR signaling, we examined the role of MAP kinase in gamma-toxin-induced IL-8 from HVECs [20,21]. Inhibition of MAP kinase via UO126, also reduced significantly the IL-8 response to HlgAB and HlgCB by >2-fold (Figure 3C,D). These data suggest that gamma-toxin-induced IL-8 production in HVECs is in part facilitated by EGFR activation of the MAP kinase signaling cascade. 

**Figure 4:**
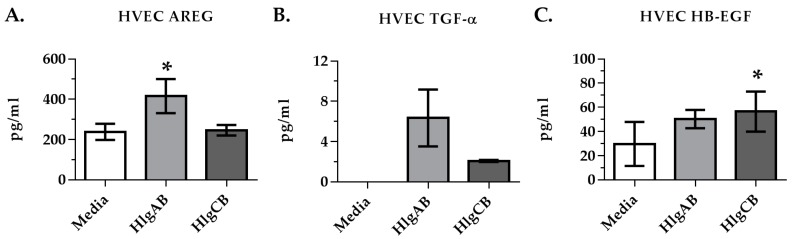
Gamma-toxin induces shedding of EGFR ligands in HVECs. Cells were incubated with HlgAB (50 µg/mL) or HlgCB (100 µg/mL) at a 1:1 molar ratio for 6 h and processed for shedding of EGFR ligands by ELISA. Data show (**A**) amphiregulin; (**B**) tumor growth factor α; and (**C**) heparin-binding epidermal growth factor mean concentrations +/− standard deviation. Asterisks indicate significant difference from media alone controls (*p* < 0.05).

**Figure 5:**
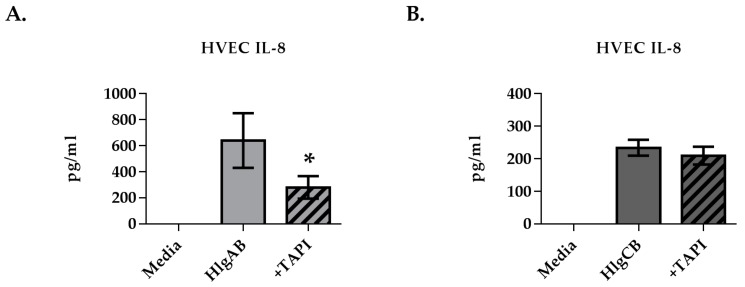
ADAM inhibition reduces HlgAB-induced IL-8 production in HVECs. HVECs were exposed to HlgAB (50 µg/mL) or HlgCB (100 µg/mL) at a 1:1 molar ratio for 6 h and processed for IL-8 production by ELISA. TAPI-1 treatment reduced IL-8 production in response to (**A**) HlgAB but not (**B**) HlgCB. Data show mean +/− standard deviation. Asterisks indicate significant difference from gamma-toxin-treated cells with no inhibitor present (*p* < 0.05).

Since EGFR signaling is required for the maximum IL-8 response to gamma-toxins in HVECs, we sought to determine if EGFR ligands were shed by HVECs upon exposure to gamma-toxins. HlgAB stimulated significantly the shedding of amphiregulin (AREG) (Figure 4A), while HlgCB stimulated significantly the shedding of heparin binding EGF-like growth factor (HB-EGF) (Figure 4C). ADAMs are cell surface proteases that mediate shedding of EGFR ligands in response to various stimuli [22]. The potential role of ADAMs in EGFR ligand shedding from HVECs in response to gamma-toxins was thus investigated using IL-8 production as a read-out of EGFR activation. The HVEC IL-8 response to HlgAB was reduced significantly through inhibition of ADAMs via TAPI-1, whereas the IL-8 response to HlgCB was not affected (Figure 5A,B). These data suggest that HlgAB stimulates shedding of EGFR ligands through an ADAM-dependent mechanism, whereas HlgCB does not.

### 2.3. HlgB Facilitates Binding of HlgA and HlgC to HVECs and Confirmation of the Presence of the Gamma-Toxin Receptors

**Figure 6:**
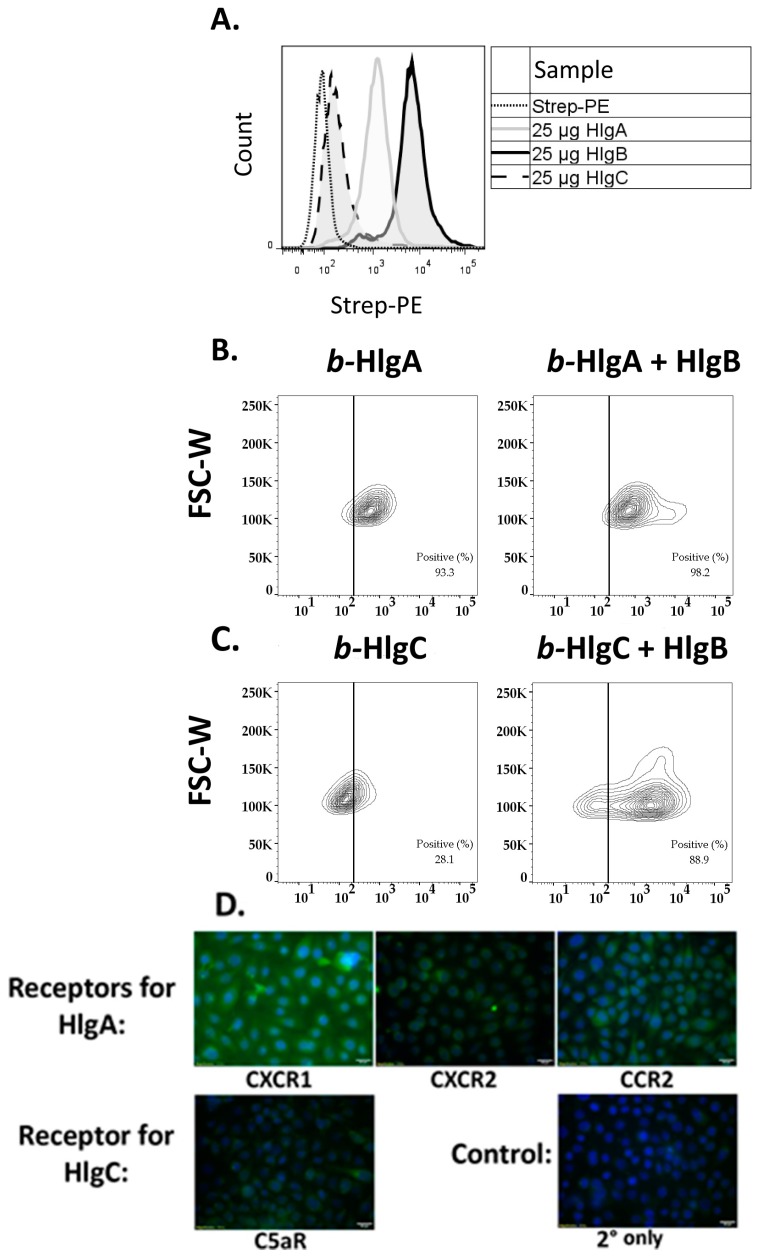
Gamma-toxin HlgB facilitates binding of HlgA and HlgC to HVECs. HVECs were incubated with 25 µg/mL of biotinylated gamma-toxin subunits at 4 °C for 30 min prior to fixation and staining. (**A**) Relative binding of individual biotinylated subunits on HVECs. Non-biotinylated HlgB significantly increased binding of biotinylated; (**B**) HlgA or (**C**) HlgC binding to HVECs (Chi Squared *T*(*X*) = 876 –* b*-Hlg*C* + HlgB and *T*(*X*) = 352 for *b*-HlgA + HlgB). Data show forward scatter width (FSC-W) versus streptavidin-phycoerythrin binding (Strep-PE). Dotted line represented cutoff for positive signal, determined from Strep-PE control. Non-specific binding of biotin negative controls was minimal <10% intensity of *b*-HlgA and *b*-HlgB bound cells (Figure S1). Data are representative of 3 experiments; (**D**) Gamma-toxin receptors’ in HVECs are shown via immunohistochemistry. Staining by the respective primary antibody/secondary antibody (fluorescein isothiocyanate, FITC) shown in green and DAPI (4',6-diamidino-2-phenylindole) in blue.

Since gamma-toxin heterodimer formation is initiated by either HlgA, HlgB or HlgC, depending on the target cell, we sought to determine the binding dynamics of gamma-toxin subunits to HVECs using flow cytometry [18,23]. We observed that biotinylated (*b*-) HlgA and *b*-HlgB subunits bound HVECs at ~93% and ~97% positive cells, respectively (Figure 6A). At equivalent doses, *b*-HlgB had higher binding intensity with ~1 × 10^4^ events per cell and *b*-HlgA with ~6 × 10^2^ events per cell. *b*-HlgC bound poorly, with 28% of cells positive and a binding-intensity of ~1.5 × 10^2^ events per cell. Non-specific binding of biotin negative-controls was minimal with <5% positive cells (Figure S1). Since HlgB bound with the greatest number of events per cell, we sought to determine if HlgB could enhance binding of *b*-HlgA or *b*-HlgC to HVECs. Upon co-incubation of non-biotinylated HlgB with either *b*-HlgA (Figure 6B) or *b*-HlgC (Figure 6C), there was a significant shift in the percent positive cells and signal intensity, with HlgA positive cells increasing to ~98% and more notably HlgC positive cells reaching 89%, indicating that HlgB drives increased binding of HlgA and HlgC to epithelial cells. The data suggest that both HlgA and HlgB bind HVECs and HlgC binding is strongly dependent on heterodimer formation with HlgB. Previous studies have identified the gamma-toxin receptors in phagocytes as CXCR1, CXCR2, and CCR2 for HlgA and C5aR for HlgC. The presence of these receptors in HVECs was confirmed with Immunohistochemistry (Figure 6D). All receptors were present, with the HlgA receptor, CXCR1, having the highest relative expression. This corresponded with HlgA binding to 93% of HVECs (Figure 6B). The receptor for HlgC, C5aR, was minimally expressed by HVECs, which corresponded with HlgC binding to 28% of HVECs (Figure 6C).

### 2.4. Gamma-Toxins-Induced IL-8 Production in Ex Vivo Tissue is Enhanced through EGFR Signaling

**Figure 7:**
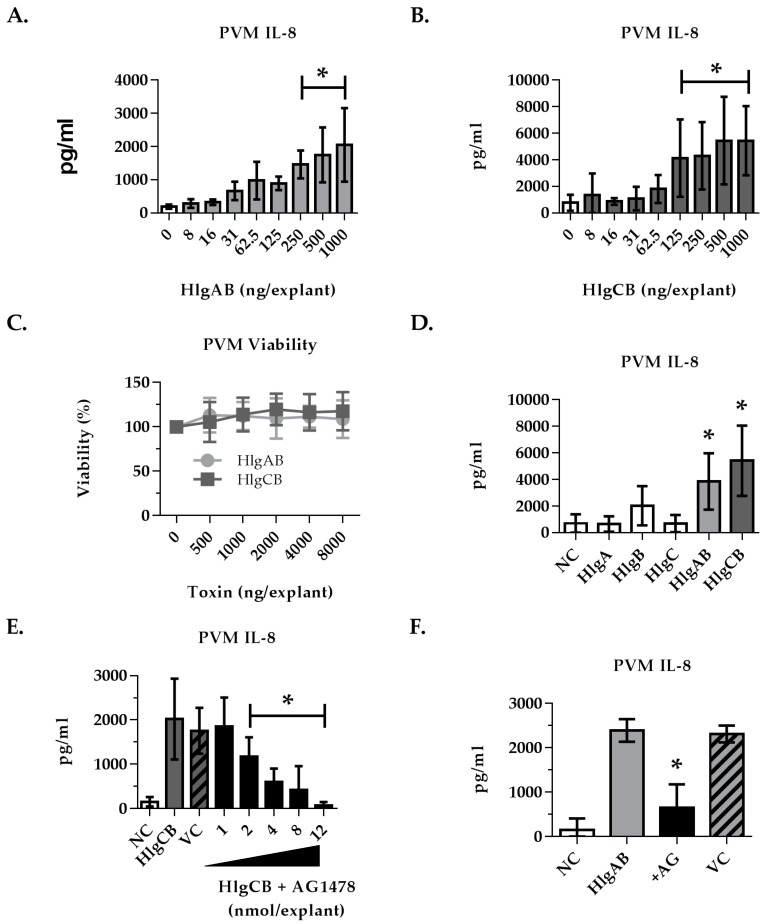
IL-8 production from porcine vaginal mucosa in response to gamma-toxin involves EGFR signaling. Ex vivo PVM was used to expand on our observations in HVECs using a complex tissue model. Explants were treated topically with gamma-toxin for 6 h prior to processing for IL-8 by ELISA. Where inhibitors were used, they were applied topically 30 min prior to gamma-toxin. IL-8 produced in response to (**A**) HlgAB and (**B**) HlgCB was dose-dependent. (**C**) Gamma-toxin toxicity on PVM was not observed; (**D**) Individual subunits (HlgA, HlgB, HlgC 1000 ng/explant) did not stimulate IL-8 at equivalent doses of HlgAB or HlgCB at a 1:1 molar ratio; (**E**) AG1478 inhibition of HlgCB (500 ng/explant)-stimulated IL-8 production in PVM was dose-dependent; (**F**) HlgAB (500 ng/explant)-stimulated IL-8 production in PVM was also reduced by EGFR inhibition by addition of AG1478 (8 nmol). NC is negative (untreated) control. AG is AG1478. VC is the AG1478 vehicle control. Asterisks indicate significant difference from media alone controls (A–D) or toxin-stimulated (E,F) (*p* < 0.05).

To investigate the hypothesis that gamma-toxins are pro-inflammatory to epithelial surfaces in a more biologically relevant model, gamma-toxins-induced IL-8 production in ex vivo PVM was measured. Both HlgAB and HlgCB significantly stimulated PVM IL-8 production in a dose-dependent manner (Figure 7A,B). In contrast to HVECs, HlgCB stimulated a more potent IL-8 response than HlgAB in PVM, generating greater than 2-fold IL-8 production at proinflammatory doses. Neither HlgAB nor HlgCB were observed to be cytotoxic to PVM at the doses tested (Figure 7C). The individual subunits did not elicit a response when applied at equivalent doses of total toxin (Figure 7D). 

As HlgAB and HlgCB stimulation of proinflammatory cytokines is enhanced through an EGFR-dependent mechanism in HVECs, we investigated the requirement for EGFR signaling in gamma-toxin-induced IL-8 production by PVM. A dose range of AG1478 (EGFR inhibitor) was applied to PVM prior to the addition of 500 ng HlgCB, the minimum dose for IL-8 saturation. AG1478 inhibited the mucosal response to HlgCB at ≥2 nmol per explant (Figure 7E). AG1478 (8 nmol per explant) also attenuated IL-8 production in response to 500 ng of HlgAB (Figure 7F). These data show that in a complex tissue model with multiple cell types, induction of IL-8 production by gamma toxins involves EGFR signaling. 

**Figure 8:**
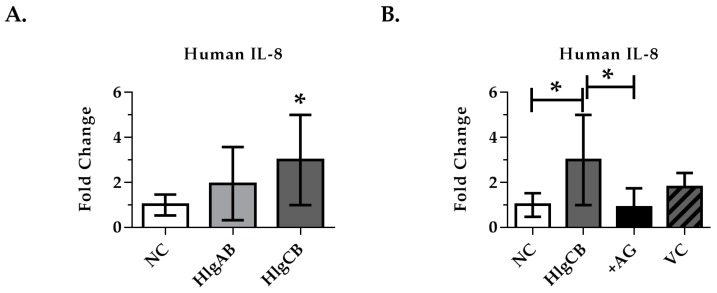
IL-8 production from human ectocervix tissue in response to gamma-toxins. Explants were treated topically with gamma-toxin for 6 h prior to analysis of IL-8 production by ELISA. AG1478 (EGFR inhibitor) was applied topically 30 min prior to gamma-toxin. (**A**) HlgCB (1000 ng/explant) increased significantly IL-8 production from human ectocervix tissue over untreated controls; (**B**) Inhibition of EGFR signaling with AG1478 (8 nmol/explant) attenuated the IL-8 production from human ectocervical tissue in response to HlgCB (1000 ng/explant). Due to high inter-person variability in background IL-8, data are represented as fold-change in IL-8 production over unstimulated controls +/− SD. Data are normalized to fold-change and combined from 3 experiments each with *n* = 3. Due to physical limitations on tissue size, the effect of the vehicle control (VC) was assayed only once with *n* = 3. NC is negative (untreated) control. AG is AG1478. Asterisks indicate a significant difference from the vehicle control (**A**) or from toxin treatment (**B**) (*p* < 0.05).

Due to the potential of species specificity, we investigated the ability of AG1478 to inhibit gamma-toxin-induced IL-8 production in ex vivo human ectocervical tissue. Human ectocervix was used to validate HVEC and porcine results instead of vaginal tissue due to availability following hysterectomies. Tissue explants obtained immediately following surgery were treated with gamma-toxin HlgAB (1000 ng) or HlgCB (1000 ng), a dose that caused robust IL-8 production in PVM (Figure 8A). Significant IL-8 production was observed with HlgCB, which could be attenuated to baseline through inhibition of EGFR signaling via AG1478 (Figure 8B). The delivery vehicle, 10% DMSO (aq) had no effect on IL-8 production in response to HlgCB. Human tissue showed high inter-person variability in background IL-8 levels, which proportionally affected the tissue response to gamma-toxin (Figure S2). However, the IL-8 response of the tissue to gamma-toxins was consistent when normalized to fold change in IL-8 over baseline. The tissue was de-identified and we were unable to control for age or co-morbidities, which may explain the high inter-person variably. This data demonstrates that HlgCB stimulates IL-8 production in an EGFR-dependent manner in human ectocervical mucosal tissue.

## 3. Discussion

*S. aureus* colonization of mucosal surfaces is common in the community with >30% of the population colonized at any time [24]. The current study identifies a novel cellular target of gamma-toxins and a potential pathway, EGFR signaling, by which gamma-toxins induce or enhance the pro-inflammatory epithelial response. This response may contribute to disruption of the vaginal mucosa, allowing *S. aureus* and its toxins access to underlying tissues and immune cells. The activities of gamma-toxins described here highlight the importance of these under-studied toxins and further elucidate the complex mechanisms by which *S. aureus* utilizes an arsenal of toxins to promote disease from colonized mucosal sites.

Gamma-toxins were independently cytotoxic and proinflammatory. The cytokines (IL-8, IL-6, TNFα and MIP-3α) produced by HVECs in response to gamma-toxins were similar to those observed in the cytokine profile induced by other *S. aureus* exotoxins, including TSST-1 and alpha-toxin [11,25]. The cytokine response elicited from epithelial cells by gamma-toxins would likely promote recruitment of neutrophils, leukocytes and dendritic cells, which may create a favorable environment for disease. *S. aureus* has a high capacity to resist killing by neutrophils and multiple toxins have been implicated in promoting their recruitment and priming, including TSST-1, Panton-Valentine leukocidin and phenol-soluble modulins [26,27]. Furthermore, over-recruitment of neutrophils during *S. aureus* infection promotes tissue damage and is speculated to aid in bacterial dissemination [28,29]. Therefore, it is possible that gamma-toxins exploit EGFR signaling to enhance the pro-inflammatory cytokine response, which promotes hyper-recruitment of neutrophils. Additionally, the cytokines produced in response to gamma-toxins promote recruitment and activation of dendritic cells, which traffic SAgs to draining lymph nodes, potentiating systemic disease [30].

Redundancies in *S. aureus* virulence factors are common. For example, *S. aureus* produces numerous SAgs, cytolysins and leukocidins, each with considerable homology within their class and overlap in targets [31,32]. However, it would appear that these redundancies extend across toxin subsets in their mechanisms to induce the innate immune system and inflammation. TSST-1 and alpha-toxin have been classically characterized as SAgs and cytolysins respectively. More recently these toxins have been identified to be disruptive to the epithelium through receptor specific interactions. Alpha-toxin interacts with ADAM10, resulting in disruption of cell–cell junctions and expression of ADAM10 is required for fatal *S. aureus* pneumonia in mice [33]. Additionally, TSST-1 stimulates a cytokine response in HVECs through an ADAM- and EGFR-dependent pathway [13]. Our findings that gamma-toxins also interact with this pathway suggests that EGFR signaling may be commonly exploited by *S. aureus* to gain access to the host through inflammation and disruption of membrane integrity or to induce a favorable innate immune response. 

Activation of the EGFR in HVECs by gamma-toxins appears to be through stimulation of shedding of EGFR ligands, specifically AREG by HlgAB and HB-EGF by HlgCB. Interestingly, TSST-1 also induces shedding of AREG and TGF-α in HVECs [13]. Shedding of EGFR ligands in response to gamma-toxins in HVECs appears to be partially mediated by ADAMs, which are known to facilitate this process [34]. However, HlgCB-induced IL-8 in HVECs was not inhibited through ADAM inhibition via TAPI-1, and the mechanism of HB-EGF shedding in HVECs by HlgCB is currently unknown. The differential inhibition of the HVEC IL-8 response to gamma-toxins suggests that each toxin stimulates an EGFR-dependent cytokine response, but only HlgAB does so through an ADAM-dependent mechanism. ADAM activation, either directly or upstream, leads to autocrine and paracrine feedback activation of the EGFR, phosphorylation of MAP kinase and activation of NF-κB, ultimately resulting in stimulation of the innate immune response [10,14,35]. Induction of EGFR-mediated cytokine production in HVECs by multiple *S. aureus* exotoxins suggests that it is an important part of *S. aureus* pathogenesis. 

Inhibiting EGFR signaling with AG1478 reduced IL-8 production from gamma-toxins in HVECs, ex vivo porcine vaginal mucosa, and ex vivo human ectocervical tissue. In our study, only inhibition of the chosen biomarker IL-8 by AG1478 was measured, and the effect on production of other cytokines is unknown. In the current study, we did not observe complete inhibition of IL-8 in HVECs exposed to gamma-toxins in the presence of the EGFR inhibitor. It is likely that inhibition of EGFR has no effect on pore formation and a general stress response may be responsible for some of the cytokine production. Currently, there are approved tyrosine kinase inhibitors that target the EGFR and the AG1478 inhibitor is a derivative of the approved drug gefitinib [36]. This raises the possibility of local inhibition of EGFR as adjunct therapy in treatment of mTSS or other mucosal infections. Such a treatment may shunt a proinflammatory response induced by and beneficial to *S. aureus*. 

Differential effects of HlgAB and HlgCB have been observed between different cell types and different species. Both HlgAB and HlgCB are able to lyse neutrophils, but only HlgCB has lytic and proinflammatory activity against human macrophages [9,31]. Gamma-toxin cell specificity in immune cells is receptor-dependent [18]. HlgAB binds the IL-8 receptors CXCR1, CXCR2 and CCR2 and HlgCB binds the complement receptor C5aR [18]. In this study, immunohistochemistry has confirmed the presence of all four receptors on the surface of HVECs, with the HlgA receptor CXCR1 having the highest relative expression. The abundance of CXCR1 versus C5aR may partially explain the differences in binding *b*-HlgA (93%) versus *b*-HlgC (28%) and the more potent lytic and pro-inflammatory effect of HlgAB in HVECs. 

In ex vivo tissue (human and porcine), HlgCB was notably more proinflammatory than HlgAB when compared to HVECs. Ex vivo PVM and human cervical tissue contain an array of cell types including resident immune cells. This may explain the differences observed in activity against cultured cells versus full thickness tissue. In addition, gamma toxin receptor(s) expression may differ in ex vivo tissue as compared to immortalized in vitro human cells. Finally, HlgCB is highly active against human macrophages, thus the enhanced potency of HlgCB over HlgAB in ex vivo tissue may be from stimulation of resident macrophages by HlgCB [9]. 

This study identifies a novel cellular target and potential mechanism of action by which gamma-toxins could enhance *S. aureus* pathogenesis through disruption of the vaginal mucosa. Furthermore, it highlights the importance of ADAMs and the EGFR in *S. aureus* mucosal pathogenesis; a pathway shared by multiple *S. aureus* exotoxins. Redundancies in *S. aureus* virulence factors have hindered vaccine development. However, if the exotoxins (superantigens and cytolysins) promote disease through a shared host pathway, it raises the potential of inhibiting the host response to the toxins as adjunctive therapy to antibiotics for *S. aureus* infections.

## 4. Materials and Methods 

### 4.1. Gamma-Toxin Purification

Gamma-toxin *hlgB* was cloned into the pET30a plasmid (Novagen, Madison, WI, USA, 69909) incorporating an *N*-terminal histidine tag and transformed into BL21 *E. coli* (Invitrogen, Waltham, MA, USA, C6000). The primers used were: 5’-GGCCGTCGACAAAGAAACTGAAAACAAT-AAATAGCTA-3’and 5’-GGCCGGATCCGGGTATAGGGGTTTTAGTATGACATC-3’. Protein expression was induced in Luria broth (kanamycin 30 µg/mL) containing 0.3 mM Isopropyl β-D-1-thiogalactopyranoside (IPTG). Bacterial pellets were lysed using lysozyme (100 µg/mL) and sonication. The supernatant was sterile-filtered and incubated with Ni-NTA resin (Invitrogen, Waltham, USA, R901) to bind the HlgB subunit. Due to poor expression in *E. coli*, *hlgA* and *hlgC* were purified from *S. aureus* as previously described [37]. Briefly, HlgA and HlgC were cloned into a plasmid (pOS1) incorporating an N-terminal histidine tag and transformed into *S. aureus* (Newman). Expression was induced with chloramphenicol (10 µg/mL) in Todd Hewitt Broth. The bacteria supernatants were sterile-filtered and incubated with Ni-NTA resin (Invitrogen, Waltham, MA, USA, R901) to bind the subunits. The proteins were eluted from the resin using an imidazole gradient. Endotoxin from HlgB preparation was removed using Pierce High Capacity Endotoxin Removal Resin per manufacture’s protocol (Pierce, Waltham, MA, USA, PI88267). Final endotoxin concentration was calculated by LAL assay and determined to be non-stimulatory in any of our models (data not shown). Biotinylated proteins were produced using EZ-Link NHS-PEG_4_-Biotinylation Kit (Thermo, Waltham, MA, USA, 21455) per manufacturer’s instructions. Briefly, protein was dialyzed into phosphate buffered saline (PBS) overnight at 4 °C then incubated with a 20 mM excess of NHS-PEG_4_-Biotin for 30 min at room temperature. Excess biotin was removed by dialyzing into PBS overnight at 4 °C.

### 4.2. Cell Culture

HVECs (ATCC CRL-2616 [38]) were grown in tissue culture-treated flasks using keratinocyte serum-free media (KSFM) (Gibco, Waltham, MA, USA, 17005-042) supplemented with bovine pituitary extract (50 µg/mL), recombinant human epidermal growth factor (0.2 ng/mL), CaCl_2_ (0.4 mM), penicillin (25 IU/mL), streptomycin (25 µg/mL), gentamicin (40 µg/mL), and amphotericin B (2.5 µg/mL); referred to as complete media. Cells were maintained at 37 °C and 7% CO_2_. For all assays, 96-well plates were seeded with HVECs at 50,000 cells/well and grown for 24 h in complete media then the media was replaced with KSFM supplemented only with 0.4 mM CaCL_2_ (referred to as minimal media) for 24 h prior to treatment.

For analysis of shedding, cytokine production and cytotoxicity, HVECs were treated in 96-well plates with 100 µl minimal media containing gamma-toxin subunits HlgAB or HlgCB at 1:1 molar ratios for 6 h at 37 °C and 7% CO_2_. After incubation, the media was assayed by ELISAs purchased from R & D Systems per manufacturer’s protocol for IL-8, IL-6, TNF-α or MIP-3α (DY208, DY206, DY210, DY360) or the EGFR ligands AREG, TGF-α, and HB-EGF (R&D Systems, McKinley, USA, DY262, DY239, DY259). Following removal of the supernatant for cytokine assays, the media was replaced with 100 µL minimal media supplemented with 20 µL MTS tetrazolium (Promega, Madison, WI, USA, CellTiter-96 G3582) and incubated for 2 h at 37 °C and 7% CO_2._ The optical density was measured and viability calculated as ((treated − 100% lysis)/(Untreated − 100% Lysis)). Measured values below the lower limit of detection were reported as measured but not considered significant, specifically IL-6 for HlgCB and TNF-α values.

For analysis of the effects of inhibitors, each inhibitor was added at twice the final concentration in 50 µL of minimal KSFM 30 min prior to the addition of gamma-toxin, HlgAB or HlgCB at 1:1 molar ratio, in a final volume of 100 µL for 6 h at 37 °C in 7% CO_2_. AG1478 (Tocris Bioscience, Bristol, UK, 1276) was used at a final concentration of 1 µM, TAPI (Enzo Life Sciences, Farmingdale, NY, USA, BML-PI134-0001) at 50 µM, and UO126 (Tocris Biosciences, 1144) at 10 µM. Inhibitor concentrations for HVECs were chosen based on previous studies [13].

For gamma-toxin binding studies, HVECs were grown as described earlier, treated with trypsin, and resuspended at 5 × 10^5^ cells/mL. Cells were treated with 25 µg/mL of the biotinylated (*b*-) *b*-HlgA, *b*-HlgB, or *b*-HlgC for 30 min on ice, the equivalent dose used for cytokine and shedding assays. Non-biotinylated HlgB at 25 µg/mL was also incubated with *b*-HlgA or *b*-HlgC. Biotin negative controls were prepared from protein negative controls and used at equal volume. Cells were washed three times with PBS and incubated with 250 ng streptavidin r-phycoerythrin (Strep-PE) (Peirce, Waltham, MA, USA, 21627) at 5 µg/mL in PBS for 20 min. The cells were fixed in 2% paraformaldehyde for 15 min on ice, washed with PBS, and analyzed by flow cytometry (FlowJo v10.3.0) for 20,000 events. Cells were gated based on forward and side scatter area. Positive selection gate for Strep-PE was defined to exclude 99% of the Strep-PE control subset. 

For immunohistochemistry, HVECs were grown as described earlier, treated with trypsin, and seeded in 8-well chamber slides with ~30,000 cells. The cells were treated with −10 °C methanol (200 μL for 5 min, then allowed to air dry. Once dried, the cells were blocked with 10% normal blocking serum in PBS (300 μL) for 20 min. Cells were incubated with 300 μL of a primary antibody (Santa Cruz Biotechnology, TX, USA) at a concentration of 4 ug/mL in PBS 1.5% normal blocking serum, SC-7303 for CXCR1, SC-7304, for CXCR2, SC-74490 for CCR2, or SC-53797 for C5aR, for 60 min then were washed in PBS three times for 5 min each. Cells were incubated with a fluorochrome conjugated secondary antibody (goat anti-mouse IgG-fluorescein isothiocyanate (FITC); SC-2010, Santa Cruz Biotechnology, TX, USA) (300 μL at 3 μg/mL) for 45 min in a dark chamber then washed three times with PBS. Cells were then stained with DAPI (Invitrogen, Waltham, MA, USA, D1306) per manufacturer’s instructions and visualized on an epi-fluorescent microscope (Olympus, Waltham, MA, USA, BX63F).

### 4.3. Porcine Tissue Procurement and Assays

Porcine vaginal mucosa (PVM) was collected and processed prior to treatment as previously described [39]. Briefly, porcine vaginal tissue was collected at time of slaughter in Roswell Park Memorial Institute (RPMI) Media 1640 (Gibco, Waltham, MA, USA, 11875-093) supplemented with 10% fetal calf serum, penicillin (50 IU/mL), streptomycin (50 mg/mL), amphotericin B (2.5 µg/mL) and gentamicin (40 µg/mL). Antibiotics were included to eliminate native flora. Within 3 h of slaughter, 5 mm biopsies were taken and excess muscle tissue trimmed. These explants were washed three times in RPMI containing antibiotics, and then incubated for 30 min at 37 °C in antibiotic-free RPMI. Following washing, explants were placed mucosal side up on PET track-etched 0.4 µm cell culture inserts (BD Biosciences) in 6-well plates above fresh antibiotic- and serum-free RPMI media with the epithelial surface exposed to air.

For cytokine analysis, PVM explants were treated topically with HlgAB or HlgCB, at 1:1 molar ratios and incubated for 6 h at 37 °C and 7% CO_2_. After treatment with gamma-toxin, biopsies were vortexed in 250 µL PBS for 4 min at maximum speed to lyse epithelial cells and release IL-8 for quantitation. The remaining connective tissue was centrifuged at 13,000 rpm for 1 min and the amount of IL-8 in the supernates determined via ELISA (R & D Systems, McKinley, USA DY535). Viability of PVM explants was determined using the Cell Growth Determination kit (Sigma, St. Louis, MO, USA, CDG1). Explants were incubated in 20 µL MTT reagent and 100 µL RPMI for 3 h at 37 °C and 7% CO_2_ then in 100 µL isopropanol. Viability of treated tissue was determined by the optical density (570 nm) of the supernatant compared to untreated controls.

For analysis of the effects of inhibitors, PVM explants were treated at −30 and −10 min with 8 nmol AG1478 (Tocris Bioscience, 1276) in 10% DMSO (aq) prior to the addition of gamma-toxin (HlgAB or HlgCB at 1:1 molar ratio). The explants were incubated for 6 h and assayed for IL-8 as described above.

### 4.4. Human Tissue

Human ectocervix was obtained from UMN BioNet Tissue Procurement post-surgery. Five- millimeter biopsies were taken from normal tissue, trimmed of excess connective tissue, washed three times in minimal KSFM, and incubated in minimal KSFM for 30 min at 37 °C in 7% CO_2_. Following washing, the biopsies were placed mucosal side up on PET track-etched 0.4 µm cell culture inserts (BD Biosciences) in 6-well plates above minimal KSFM with the epithelial surface exposed to air. The explants were treated at -30 and -10 min with 8 nmol AG1478 (Tocris Bioscience, Bristol, UK, 1276) in 10% DMSO (aq) prior to the addition of gamma-toxin (HlgAB or HlgCB at 1:1 molar ratio) for 6 h at 37 °C in 7% CO_2_. Following treatment, the biopsies were vortexed in 250 µL PBS for 4 min at maximum speed to lyse epithelial cells and release IL-8 for quantitation. The remaining connective tissue was centrifuged at 13,000 rpm for 1 min and the supernatant assayed by ELISA for IL-8 using manufactures protocol (R & D, DY208). Since the tissue was obtained de-identified, we had no knowledge of the age or other co-morbidities which could affect baseline IL-8 levels and due to high inter-person variability in baseline IL-8 the data were normalized to fold change. 

### 4.5. Statistical Analysis

All data are combined averages of 3 experiments with 3 replicates (*N* = 3, *n* = 3) unless otherwise noted. Statistical differences were analyzed used One-Way ANOVA with either Dunnett’s or Bonferroni’s multiple comparison test. 

### 4.6. Ethics Statement

Porcine vaginal tissue was obtained from animals being slaughtered for human consumption by the Andrew Boss Meat Science Laboratory at the University of Minnesota and is Institutional Animal Care and Use Committee (IACUC) exempt. Human tissue was obtained from the Tissue Procurement Center at the University of Minnesota. Tissue was de-identified and determined by the Institutional Review Board (IRB) to not meet the regulatory definition of research with human subjects.

## Supplementary Materials

The following are available online at www.mdpi.com/2072-6651/9/7/202/s1, Figure S1: Non-specific binding of biotin negative controls was minimal. HVECs were incubated with equal volumes of biotinylated negative-control protein preparations, as used in Figure 6, prior to fixation and staining. (A) Binding of *b*-HlgA and *b*-HlgC vs. Negative Control. (B) Binding of *b*-HlgB vs. Negative Control. Figure S2: IL-8 production from human ectocervix tissue in response to gamma-toxins. Explants were treated topically with gamma-toxin for 6 h prior to analysis of IL-8 production by ELISA. HlgAB or HlgCB (1000 ng/explant) increased significantly IL-8 production from human ectocervix tissue over negative (untreated) controls (NC). Each data point shows averages from an experiment of *n* = 3 with the control and experimental treatment connected by line for each corresponding treatment. Untreated controls showed higher variability in baseline IL-8. Data suggests an increased response, represented by an increased slope, correlating to baseline IL-8 of untreated controls.Data for figures presented in this paper have been deposited online at the University of Minnesota Digital Conservancy: http://dx.doi.org/10.13020/D6QG61.

## Abbreviations

The following abbreviations are used in this manuscript:

Hlg	Gamma-toxin
*b*-Hlg	Biotinylated Gamma-toxin
NC	Negative Control
VC	Vehicle Control
HVEC	Human Vaginal Epithelial Cells
PVM	Porcine Vaginal Mucosa
TSS	Toxic Shock Syndrome
mTSS	Menstrual Toxic Shock Syndrome
EGFR	Epidermal Growth Factor Receptor
ADAM	A Disintegrin and Metalloprotease
PBS	Phosphate Buffered Saline
TBS	Tris-Buffered Saline
DAPI	4’,6-Diamidino-2-Phenylindole
IL-8	Interleukin-8
IL-6	Interleukin-6
TNF-α	Tumor Necrosis Factor Alpha
MIP-3 α	Macrophage Inflammatory Protein-3
AREG	Amphiregulin
TGF- α	Tumor Growth Factor Alpha
HB-EGF	Heparin-Binding Epidermal Growth Factor
Strep-PE	Streptavidin r-Phycoerythrin
DMSO	Dimethyl Sulfoxide
FSC-W	Forward Scatter Width
IPTG	Isopropyl β-D-1-thiogalactopyranoside
KSFM	Keratinocyte Serum-Free Medium
RPMI	Roswell Park Memorial Institute Medium
