# Supplementary material for: Epidermal Growth Factor Receptor Signaling Enhances the Proinflammatory Effects of Staphylococcus aureus Gamma-Toxin on the Mucosa
Source: Toxins (Basel). 2017 Jun 28;9(7):202. doi: 10.3390/toxins9070202 (PMC5535149; doi:10.3390/toxins9070202)
Supplement: Supplementary file 1 [file toxins-09-00202-s001.pdf]

Supplementary Materials: Epidermal Growth Factor Receptor Signaling Enhances the Proinflammatory Effects of *Staphylococcus aureus* Gamma-Toxin on the Mucosa

Aaron N. Gillman, Laura M. Breshears, Charles K. Kistler, Patrick M. Finnegan, Victor J. Torres, Patrick M. Schlievert and Marnie L. Peterson

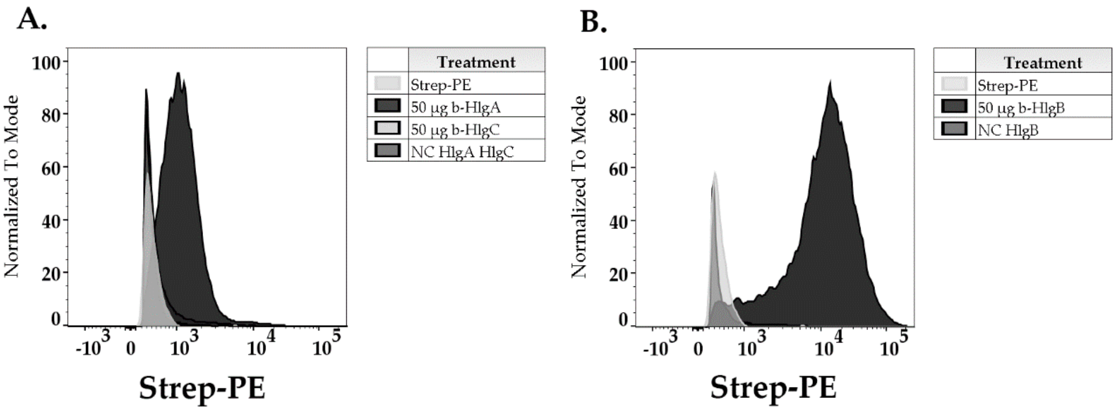

Figure S1. Non-specific binding of biotin negative controls was minimal. HVECs were incubated with equal volumes of biotinylated negative-control protein preparations, as used in Figure 6, prior to fixation and staining. (**A**) Binding of *b*-HlgA and *b*-HlgC vs. Negative Control. (**B**) Binding of *b*-HlgB vs. Negative Control.

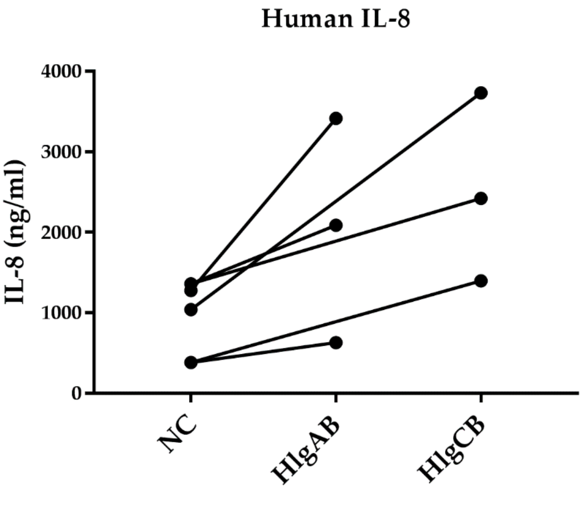

Figure S2. IL-8 production from human ectocervix tissue in response to gamma-toxins. Explants were treated topically with gamma-toxin for 6 h prior to analysis of IL-8 production by ELISA. HlgAB or HlgCB (1000 ng/explant) increased significantly IL-8 production from human ectocervix tissue over negative (untreated) controls (NC). Each data point shows averages from an experiment of *n* = 3 with the control and experimental treatment connected by line for each corresponding treatment. Untreated controls showed higher variability in baseline IL-8. Data suggests an increased response, represented by an increased slope, correlating to baseline IL-8 of untreated controls.
